# Soluble Erythropoietin Receptor Contributes to Erythropoietin Resistance in End-Stage Renal Disease

**DOI:** 10.1371/journal.pone.0009246

**Published:** 2010-02-16

**Authors:** Eliyahu V. Khankin, Walter P. Mutter, Hector Tamez, Hai-Tao Yuan, S. Ananth Karumanchi, Ravi Thadhani

**Affiliations:** 1 Department of Medicine, Beth Israel Deaconess Medical Center and Harvard Medical School, Boston, Massachusetts, United States of America; 2 Department of Medicine, Massachusetts General Hospital and Harvard Medical School, Boston, Massachusetts, United States of America; 3 Howard Hughes Medical Institute, Beth Israel Deaconess Medical Center and Harvard Medical School, Boston, Massachusetts, United States of America; All India Institute of Medical Sciences, India

## Abstract

**Background:**

Erythropoietin is a growth factor commonly used to manage anemia in patients with chronic kidney disease. A significant clinical challenge is relative resistance to erythropoietin, which leads to use of successively higher erythropoietin doses, failure to achieve target hemoglobin levels, and increased risk of adverse outcomes. Erythropoietin acts through the erythropoietin receptor (EpoR) present in erythroblasts. Alternative mRNA splicing produces a soluble form of EpoR (sEpoR) found in human blood, however its role in anemia is not known.

**Methods and Findings:**

Using archived serum samples obtained from subjects with end stage kidney disease we show that sEpoR is detectable as a 27kDa protein in the serum of dialysis patients, and that higher serum sEpoR levels correlate with increased erythropoietin requirements. Soluble EpoR inhibits erythropoietin mediated signal transducer and activator of transcription 5 (Stat5) phosphorylation in cell lines expressing EpoR. Importantly, we demonstrate that serum from patients with elevated sEpoR levels blocks this phosphorylation in *ex vivo* studies. Finally, we show that sEpoR is increased in the supernatant of a human erythroleukaemia cell line when stimulated by inflammatory mediators such as interleukin-6 and tumor necrosis factor alpha implying a link between inflammation and erythropoietin resistance.

**Conclusions:**

These observations suggest that sEpoR levels may contribute to erythropoietin resistance in end stage renal disease, and that sEpoR production may be mediated by pro-inflammatory cytokines.

## Introduction

Management of anemia with erythropoietin in patients with renal failure on dialysis has significantly changed clinical practice in nephrology. The widespread clinical use of recombinant erythropoietin has reduced the requirement for blood transfusions and is associated with improved left ventricular hypertrophy [1] as well as quality of life outcomes in dialysis patients [2]–[4]. Recent clinical studies, however, have suggested an excess of cardiovascular events in patients requiring elevated doses of erythropoietin [5]–[7] and a trend toward excess mortality in dialysis patients with higher hemoglobin targets [8]. It is not clear if this excess risk is related to erythropoietin dose or an absolute rise in hemoglobin concentration. As a result there is significant debate about optimal erythropoietin dose and hemoglobin targets. Nevertheless, erythropoietin therapy still remains a cornerstone in the management of anemia in chronic dialysis patients.

Erythropoietin is protein hormone produced by the kidney in response to hypoxia. Erythropoietin binds the membrane bound erythropoietin receptor (EpoR) located on erythroblasts in the bone marrow. Following receptor binding, an intracellular signaling cascade leads to the transcription of anti-apoptotic genes. This results in production of new red blood cells and improvement in anemia. While most patients respond well to erythropoietin, a subset of patients are considered resistant to erythropoietin, requiring 25–100% higher doses than the average patient to maintain acceptable hemoglobin levels [9]–[11]. This lack of response termed “Erythropoietin Resistance” or “Erythropoietin Hyporesponsiveness” is multifactorial but may be related to iron stores (both absolute and effective), renal osteodystrophy, and inflammation [9]–[11]. Generally patients who receive in excess of 400 IU/kg per week of erythropoietin and fail to achieve target hemoglobin are considered resistant although there is clearly a spectrum of erythropoietin sensitivity [9]. It remains clinically challenging to accurately predict which patients will be resistant to erythropoietin prior to initiating erythropoietin therapy. Chronic inflammation seems to play a role in some patients and there is evidence that inflammatory cytokines may down regulate the bone marrow response to erythropoietin [10], [11]. In such cases there may be functional iron deficiency related to inadequate delivery of iron to the erythroid marrow in the face of adequate iron sores in the reticuloendothelial system [12]. Iron delivery may be inhibited by inflammatory cytokines and hormones such as hepcidin which may play a role in renal anemia [13]–[15]. In addition, markers of erythropoiesis such as the soluble transferrin receptor (sTfR) may be useful in determining appropriate iron management strategies [16]–[19].

EpoR is a membrane-bound receptor present in erythroblasts and is a member of the cytokine superfamily of type 1 transmembrane proteins [20], [21]. Erythropoietin binding results in phosphorylation of intracellular messengers such as Stat-5 that bind tyrosine residues, become phosphorylated and translocate to the nucleus to initiate gene transcription [22]. Other intracellular signaling cascades may be activated as well including phosphoinositide 3-kinase, IkappaB kinase, and heat shock protein 70 [22], leading to transcription of anti-apoptotic factors [22]. Importantly, alternative mRNA splicing produces a soluble form of EpoR (sEpoR) that is present in human blood [23]–[25]. Soluble EpoR consists of the extracellular domain only and has a reported molecular weight of between 27 and 34 kDa with differences presumed related to glycosylation, although sEpoR protein has not yet been purified from serum or sequenced [23], [25]–[27]. It is also possible that there is more than one form of circulating sEpoR [28] as more than one splice variant have been identified [29]. The function of sEpoR is unknown, but levels are thought to correlate with the amount of erythropoiesis, raising the possibility of a physiologic role for this soluble receptor [25], [30]. Soluble EpoR has been identified in human serum and plasma as well as a number of tissues including brain, liver, spleen, kidney, heart and bone marrow [30], [31]. It has also been identified as a secreted product from several cancer cell lines [29]. It is well known that soluble receptors often play an important role in cytokine signaling by stabilizing their ligand, changing concentrations of active ligand or by altering the interaction between endogenous cytokine and membrane bound ligand [32], [33]. Although it has been shown that secreted sEpoR is able to bind erythropoietin, the role of circulating sEpoR in humans remains largely unknown [25], [30], [34].

We hypothesized that circulating sEpoR competes with erythropoietin for receptor binding and that elevated levels of sEpoR at initiation of hemodialysis portend increased erythropoietin dose requirements needed to sustain target hemoglobin levels. Furthermore we hypothesized that sEpoR production may be mediated by inflammatory cytokines present in the uremic milieu.

## Materials and Methods

### Immunoprecipitation

Human serum samples from ESRD subjects (approximately 1 ml) were mixed in a 1∶1 ratio with IP lysis buffer (150 mM NaCl, 10 mM Tris pH 7.5, 1 mM EDTA, 50 nM NaF, 1%Triton X, 0.5% NP40, Na Orthovanadate 200 µM, PMSF 10 µg/ml, protease Inhibitor and phosphatase inhibitor at 4°C. Primary antibody directed against EpoR (R&D Systems, AF-322-PB goat anti-human polyclonal or R&D Systems, MAB307 mouse anti-human monoclonal (approximately 0.5 µg/reaction) was added to each tube and incubated over night at 4°C.followed by precipitation with Protein G coated magnetic beads, then pelleted and supernatant discarded. Beads were washed three times with lysis buffer. Bound proteins were eluted with 2X loading buffer (Laemmli's SDS–sample buffer 4x reducing, Boston BioProducts No. BP-110R) according to manufacturer's recommendations. The supernatant was retained and the beads discarded. Equal amounts of eluted proteins were separated on an acrylamide 4–12% gradient gel. Protein was transferred to PDVF using a semi-dry transfer and western blotting performed using either of the two EpoR antibodies at concentrations of 1/1,000 (R&D Systems, MAB307 mouse anti-human monoclonal or R&D Systems, AF-322-PB goat anti-human polyclonal).

### Protein Sequencing/Mass Spectrometry

Immunoprecipitation was performed as described above with the exception that 30 ml of pooled serum from uremic subjects were used. Denaturing acrylamide gels were stained with 0.1% Coomassie Brilliant Blue. A band of appropriate size and a section of gel for negative control were cut out and rinsed in 1% acetonitrile. Gel fragments were subject to trypsin digest and mass spectrometry at the Beth Israel Deaconess proteomics core laboratory. Briefly, the samples were treated with trypsin (1∶100) and resuspended in 1% trifluoroacetic acid and injected it into a CapLC (Waters) high performance liquid chromatography instrument. The peptides were separated using a 75 µm Nano Series column (LC Packings) and analyzed them using a Qstar XL MS/MS system. The peptides were searched using the Mascot search engine (Matrix Science) against the human protein database NCBInr.

### sEpoR ELISA

ELISA testing of serum samples and cell culture supernatants was performed using an Erythropoietin Receptor DuoSet ELISA (DY307, R&D Systems, Minneapolis, MN). On the day of the ELISA assay, frozen serum or cell culture supernatant samples were thawed at room temperature and 100 µl (100%) serum was added to each well. A standard curve was generated using serial dilutions of recombinant sEpoR (R&D Systems 307-ER/CF) at concentrations ranging from 4 ng/ml to 62.5 pg/ml in Reagent Diluent (1% BSA/Phosphate Buffered Saline, R&D Systems DY995). All reactions were carried out at room temperature. Plates were coated overnight with primary mouse anti-human EpoR antibody in PBS 1 µg/mL (100 µl/well). On the day of the assay each well was washed three times with wash buffer (0.05% Tween 20/Phosphate Buffered Saline, R&D Systems WA126) 400 µl/well using a Columbus Pro washer (Tecan, Inc). Reagent Diluent 300 µl/well was added and plates incubated for 1 hour. Plates were then washed three times with wash buffer and 100 µl standard or sample was added to each well. Plates were incubated for 4 hours on a table top shaker and washed three times with 400 µl/well Wash Buffer. Secondary biotinylated mouse anti-human EpoR antibody 500 ng/ml (100 µl/well) was added and plates were incubated for 2 hours on a table top shaker. Plates were washed again with Wash Buffer three times and Streptavidin conjugated to Horseradish-Peroxidase (100 µl/well) was added for 20 minutes. Plates were washed three times in wash buffer and color reagent containing H_2_0_2_ and tetramethylbenzidine (100 µl/well) was added (R&D Systems DY993). Plates were incubated for 20 minutes and Stop Solution 2N H_2_S0_4_ 50 µl (R&D Systems DY994) was added to each well. Optical density was measured using a Microplate Reader (BioRad, Model 660). Optical density at 550 nm was subtracted from optical density at 450 nm and serum concentrations were determined using a standard curve generated by a quadratic plot of the standard sample concentrations versus the measured optical density. We ran the assay in duplicate for all samples with the exception of 18 serum samples which were measured only once as we were limited by sample volume. The operator was blinded to the clinical data.

The antibodies used in the R&D Systems DuoSet ELISA have proprietary epitopes that target the extracellular region of the receptor (AA 25 to 225). The ELISA was able to detect two alternative recombinant sEpoRs (Sigma-Aldrich E0643 and R&D Systems 963-ER) in addition to the sEpoR (R&D Systems 307-ER/CF) used to generate the standard curve. We calculated intra and inter-assay coefficients of variance using 3 serum samples run in triplicate on three separate plates. The mean intra assay coefficient of variance for the three samples was 4.71% range (1.10% to 9.49%) and the mean inter assay coefficient of variance was 4.74% range (1.20% to 9.27%). We also tested for erythropoietin interference by spiking both serum and control wells with recombinant erythropoietin up to 10,000 mU/ml and observed no significant alteration in EpoR measurements suggesting the assay measures total sEpoR rather than free sEpoR.

### BaF3/EpoR Stat-5 Signaling Assay

BaF3/EpoR cells stably expressing the EpoR receptor were obtained from Dr. Laurie Feldman, Beth Israel Deaconess Medical Center [35], [36]. Cells were plated at 500,000 cells per well in a 24 well dish and grown overnight in RPMI with 1% FCS. Media was replaced with serum free media and incubated for 12 hours. Vehicle, recombinant erythropoietin (Amgen), human serum, and recombinant receptor (R&D Systems) was added to wells to a total volume of 500 µl (50 µl serum per well) with erythropoietin 50 mU/ml and recombinant receptor. Cells were incubated for 10 minutes at 37°, and immediately lysed in RIPA buffer with protease inhibitors and samples frozen at −80°C for further analysis by western blots using phospho-Stat-5 and Stat-5 antibodies (Stat-5 antibody (No. 9363), phospho-Stat-5, Thyr694, antibody (No. 9351) both from Cell Signaling Technology, Danvers MA.

### Statistical Considerations for *In Vitro* Data

Standard statistical analysis was performed on all data. Individual values were collated as means +/− S.E.M. Comparisons between multiple groups were by a two way ANOVA test followed by a Mann-Whitney nonparametric test or a paired t test when appropriate. Statistical significance was considered if p <0.05.

### Human Subjects

In order to test if sEpoR levels at the initiation of chronic hemodialysis are correlated with subsequent erythropoietin requirements, we selected consecutive chronic hemodialysis (CHD) patients from the Accelerated Mortality on Renal Replacement (ArMORR) cohort study which has been described in detail [37]–[41]. This study was approved by the Institutional Review Board of the Massachusetts General Hospital and informed consent was waived by the Institutional Review board as only archived samples were used in this study. ArMORR includes 10,044 subjects who initiated CHD at any one of the 1056 U.S. dialysis centers operated by Fresenius Medical Care North America (Waltham, MA) between 2004 and 2005. All the subjects underwent 1 year follow-up except for those who died (15%), underwent kidney transplant (3%), recovered renal function (4%), or transitioned to a dialysis unit outside the Fresenius Medical Care North America system before completing 1 year of hemodialysis treatment (12%).

Within this cohort we targeted 500–1000 consecutive patients with adequate baseline blood samples and who survived at least 6 months so as to examine erythropoietin requirements during this time period. This sample size was chosen based on our previous studies examining baseline levels of other parameters and subsequent outcomes [37], [39], and because no prior human data were available to provide us with an estimate of effect size and appropriate sample size to achieve adequate power. Of these, 697 subjects representing over 400 CHD units in the U.S. had adequate remnant volumes of baseline samples with complete erythropoietin dosing information (amount of erythropoietin administered at each dialysis session). Specifically, each subject had a blood sample collected within 14 days of initiating CHD and each subject was new to CHD. No subject had ever undergone CHD or peritoneal dialysis in the past, and no subject had ever undergone a renal transplantation prior to enrollment or recovered renal function during the study period. We measured sEpoR levels by ELISA assay and the distribution plot suggested that approximately 5% of ESRD subjects had high circulating levels of EpoR (>2 standard deviations from the mean). We therefore divided the 697 patients into two groups based on sEpoR levels at CHD start. The High sEpoR group had sEpoR levels ≥800 pg/ml (n = 36), and the Low sEpoR group (n = 661) included the remainder.

### Statistical Considerations for Clinical Study

Categorical and continuous variables were examined using standard univariate tests for comparison. Longitudinal analysis was used to test the group (sEpoR Low vs. High) differences over time. Cumulative erythropoietin dose was categorized as follows: mean weekly dose administered between days 0 and 14 (14d) after initiating CHD; mean weekly dose administered between days 14 and 90 (90d); and mean weekly dose administered between days 90 and 180 (180d). Mean hemoglobin levels were categorized in a similar fashion. We performed repeated measures ANOVA to examine within and between sEpoR group comparisons. The interaction between time and sEpoR group status was tested to determine whether the relationship with erythropoietin requirements and hemoglobin levels differed between groups over time. Logistic and linear regression analyses were used to adjust for potential confounders. All analyses were performed using SAS version 9.1 for Windows (SAS Institute Inc., Cary, NC, USA). Two-sided P values <0.05 were considered statistically significant.

### Cell Culture, Stimulation of K562 Cells and Assay of sEpoR in Supernatants

K562 cells (ATCC, Virginia, CCL-243) were maintained in IMDM media +10% FCS were plated at 500,000 cells per well in a 12 well plate without serum. At time zero cells were exposed to vehicle, phorbol ester 500 nM and 1000 nM, TNF-α 50 ng/ml, IL-6 10 ng/ml, and IL-8 10 ng/ml, in triplicate for 48 hours. At the end of 48 hours cells were pelleted at 4°C. The supernatant was collected and stored at −80°C. EpoR ELISA was performed on the supernatants as described above. Protein assay was performed as follows using BCA protein assay (Pierce, Thermo Fisher Scientific, Rockford IL) according to manufacturer's standard protocol.

### IL-6 Levels

Serum IL-6 was measured using a commercially available ELISA kit (Catalog # DY6050, R&D Systems, MN) and manufacturer's instructions were followed. All 32 subjects with high sEpoR (>800 pg/ml) who had remaining serum available and an equal number of randomly chosen subjects with low sEpoR (≤62.5 pg/ml) were included for this substudy. Data are depicted as mean IL-6 levels in pg/ml +/− S.E.M for both groups.

## Results

### sEpoR Is Present in the Serum of Dialysis Patients

**Figure 1:**
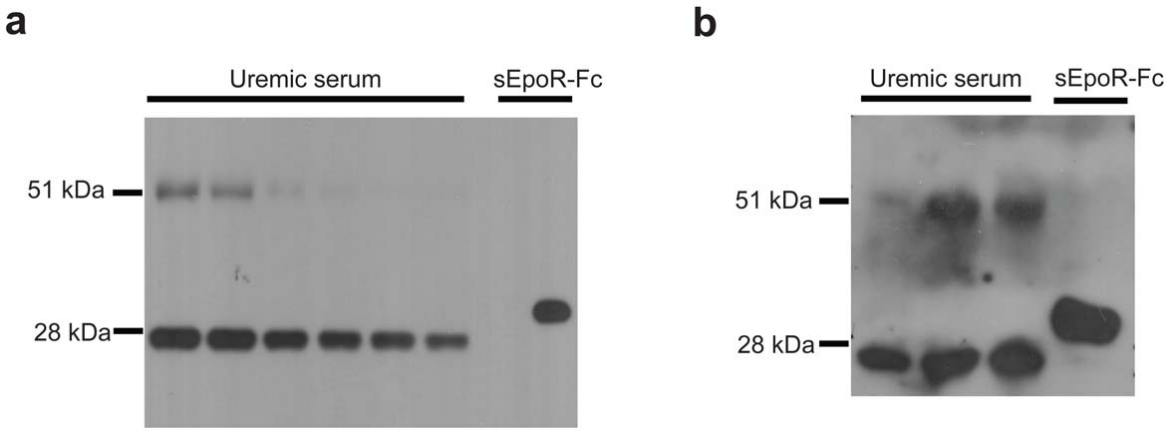
sEpoR characterization in uremic serum. **1a**. Soluble EpoR is detectable in serum from dialysis patients by western blot. Human serum was subjected to immunoprecipitation with goat anti-human erythropoietin receptor antibody (R&D Systems, AF-322-PB) followed by western blotting with mouse monoclonal anti-human erythropoietin receptor (R&D Systems, MAB307). Both antibodies recognize the extracellular domain of the receptor. Lanes 1–6 are serum from 6 representative dialysis patients, lane 7 is blank and lane 8 is recombinant sEpoR (Sigma Aldrich E0643, Saint Louis MI). Shown in the serum samples is a band of expected molecular weight of approximately 27 kDa. The control sEpoR with Fc tag is consistent with the manufacturers reported molecular weight of 32 kDa. **1b**. Soluble EpoR is also detected using the same dialysis patient serum samples by performing immunoprecipitation in reverse order. In this experiment immunoprecipitation was done with mouse monoclonal anti-human erythropoietin receptor (R&D Systems, MAB307) followed by western blotting with goat anti-human erythropoietin receptor (R&D Systems, AF-322-PB). Lanes 1 to 3 are serum from 3 dialysis patients, and lane 4 is recombinant sEpoR-Fc (Sigma, 307) as positive control.

We initially attempted to characterize sEpoR in uremic serum using two different antibodies by western blot and were unable to detect any specific band that was consistent with the expected size of sEpoR. However, immunoprecipitation with goat anti-human erythropoietin receptor followed by western blotting with mouse anti-human erythropoietin receptor revealed a clear band of approximately 27 kDa consistent with the expected size of sEpoR in 6 representative dialysis patient samples (**Figure 1a**). A band of similar size was also detected when immunoprecipitation and western blotting was performed with the same antibodies in reverse order (**Figure 1b**). Purification of the 27 kDA protein from sera of uremic subjects and analysis by mass spectrometry revealed at least 2 different peptides (GPEELLCFTERL and YEVDVSAGNGAGSVQR) corresponding to the N-terminal extracellular region of the EpoR. Taken together with the immunoprecipation studies (**Figure 1a, 1b**), these data suggest that dialysis subjects have sEpoR in their serum.

### Elevated sEpoR Levels at Initiation of Dialysis Predict Subsequent Erythropoietin Dose

**Figure 2:**
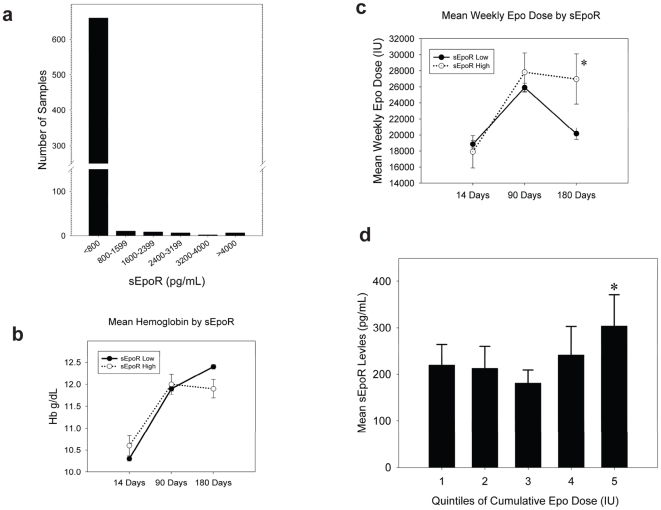
Relationship of sEpoR and erythropoietin dosage in human ESRD subjects. **2a.** This figure shows the distribution of sEpoR levels as measured by ELISA in our sample of 697 dialysis patients. **2b**. Erythropoietin dose over time for the Low and High sEpoR groups. Erythropoietin dose at the start of dialysis (Days 0 to14) was the same in both groups, 17,908 IU/week in the High sEpoR group vs. 18,851 IU/week in the Low sEpoR group. Between days 14 and 90 the High group received 27,819 IU/week vs. 25,906 IU/week in the Low group. By days 90 and 180 the High sEpoR group required a significantly greater dose of erythropoietin then the Low sEpoR group (26,977 IU/week vs. 20,173 IU/week, p = 0.038). It is notable that the average dose rose in both groups over the period 14 to 90 days but then fell again over the 90 to 180 day period as might be expected in patients starting erythropoietin therapy. **2c**. Mean hemoglobin was compared for the Low and High sEpoR groups at three time points (Days 0 to14, 14 to 90 and 90 to 180). Hemoglobin at the start of dialysis (Days 0-14) was not statistically different between the High and Low sEpoR groups (10.5 vs. 10.3 mg %). Between 90 and 180 day there was a trend toward lower hemoglobin in the High sEpoR group 12.4 vs. 11.9 that approached statistical significance (p = 0.054). This suggests that even though both groups likely had identical hemoglobin targets, the high sEpoR group may have impaired erythropoietin response even with significantly higher erythropoietin dose. **2d**. The cohort was divided according to quintiles of cumulative EPO dose during the study period, and within each quintile examined the mean sEpoR level. The mean sEpoR values according to quintile were: Quintile 1: 220±44 pg/ml; Quintile 2: 213±47 pg/ml; Quintile 3: 181±28 pg/ml; Quintile 4: 242±61 pg/ml; Quintile 5: 304±67 pg/ml, p value for trend  = 0.038. The mean cumulative Epo doses in units according to quintile were: Quintile 1: 209,004±4863, Quintile 2: 347,019±2655, Quintile 3: 459,719±3362, Quintile 4: 622,460±5334, Quintile 5: 1,077,666±25,576.

We measured sEpoR levels by ELISA in representative group of 697 incident dialysis patients. The distribution of sEpoR levels is shown in **Figure 2a**. Most of the patients had low levels of sEpoR (less than or equal to 100 pg/ml). However, a subset of patients demonstrated significantly elevated values. The distribution plot suggested that approximately 5–6% of end-stage renal disease (ESRD) subjects had high circulating levels of EpoR (>2 standard deviations from the mean), and the subjects were divided accordingly The mean levels of sEpoR at the initiation of chronic hemodialysis were 2437±1299 pg/ml in the *High* group (n = 36) and 112±111 pg/ml in the *Low* group (n = 661) (p<0.001). Median values were 2147 pg/ml (inter-guartile range 1400–3445) and 69 pg/ml (inter-quartile range 62.5–101), respectively.

**Table 1** shows the baseline characteristics of the Low and High sEpoR groups. We found no difference between the two groups with respect to age, race, gender, body mass index (BMI), or blood pressure. Importantly ferritin, iron saturation and hemoglobin were not different. In addition correlations between baseline sEpoR and ferritin, iron saturation, and hemoglobin levels were also not significant (p>0.05). Erythropoietin resistance has been associated with osteodystrophy and elevated parathyroid hormone (PTH) [9], [10], but PTH was also not different between the groups, and the correlation with PTH in the entire group was not significant as well (r = −0.03, p = 0.33). Bone biopsies were not available for these subjects to confirm osteodystrophy. Hypertension as an etiology of ESRD was slightly over represented in the Low sEpoR group. Nevertheless, standard clinical parameters did not appear to be associated with sEpoR levels.

**Table 1:** 

	sEpoR Low	sEpoR High	p-value
N	661	36	
Age (Years)	67±13	68±15	0.76
Female (%)	51	36	0.09
Race (%)			0.30
White	63	72	
Black	33	28	
Other	4	0	
aBMI (kg/m2)	26±6	27±7	0.66
Etiology of renal failure (%)			0.03
Diabetes Mellitus	47	47	
Hypertension	37	22	
Glomerulonephritis	8	8	
Polycystic Kidney Disease	2	8	
Other	6	15	
Vascular Access			0.76
Fistula	22	20	
Graft	13	17	
Catheter	65	63	
Systolic blood pressure (mmHg)	144±22	140±18	0.81
Diastolic blood pressure (mmHg)	72±13	71±10	0.31
bPTH (bio-intact; pg/ml)	297±302	278±268	0.81
Calcium (mg/dl)	8.5±0.7	8.7±0.6	0.31
Phosphorus (mg/dl)	4.5±1.5	4.3±1.8	0.46
Ferritin (ng/ml)	297±512	318±273	0.25
Iron saturation (%)	20±10	18±8	0.25
White blood cell count (cells/µl)	8±3	8±3	0.69
Platelets (cells/dl)	239±94	211±81	0.81

Results are expressed as mean ± standard deviation.

a BMI–body mass index.

b PTH–parathyroid hormone.

To determine if sEpoR levels at the initiation of chronic dialysis are associated with subsequent erythropoietin dose, we compared longitudinal weekly erythropoietin administration in our Low and High sEpoR groups (**Figure 2b**). Erythropoietin administration not only differed with time (P<0.001), but also according to sEpoR status by 90 and most notably by 180 days following hemodialysis initiation (P = 0.038). We next examined respective mean hemoglobin levels over the same time period because erythropoietin resistance has been associated with increasing erythropoietin administration in the face of lower hemoglobin levels [42]. By day 180, the Low sEpoR group had slightly higher hemoglobin levels despite receiving lower weekly erythropoietin doses (**Figure 2c**). Next, rather than categorize the cohort according to baseline sEpoR levels, we examined the cumulative erythropoietin administration as measure of erythropoietin resistance suggested by previous investigators [43]. The mean cumulative erythropoietin administration over 180 days following hemodialysis initiation was 542,102±330,308 IU. We categorized the entire cohort into quintiles of cumulative erythropoietin administration and within each quintile examined the mean baseline sEpoR levels (**Figure 2d**). Those that received higher cumulative erythropoietin doses over the study period had progressively higher baseline sEpoR levels. We then performed logistic regression analyses to determine the independent baseline variables associated with the highest cumulative erythropoietin administration (Quintile 5). In addition to High vs. Low sEpoR levels, we adjusted for characteristics previously linked with erythropoietin administration including age, race, sex, cause of end-stage renal failure (diabetes, hypertension, glomerulonephritis, other), and baseline exposures such as arterio-venous access (fistula, graft, catheter), weight, serum ferritin, transferrin saturation, and PTH levels [42]–[44]. The risk for requiring the highest erythropoietin administration in the High vs. Low sEpoR groups was approximately 3-fold higher (OR 2.8, 95% CI 1.3–6.4).

### sEpoR Blocks EPO Mediated Signaling *In Vitro*

**Figure 3:**
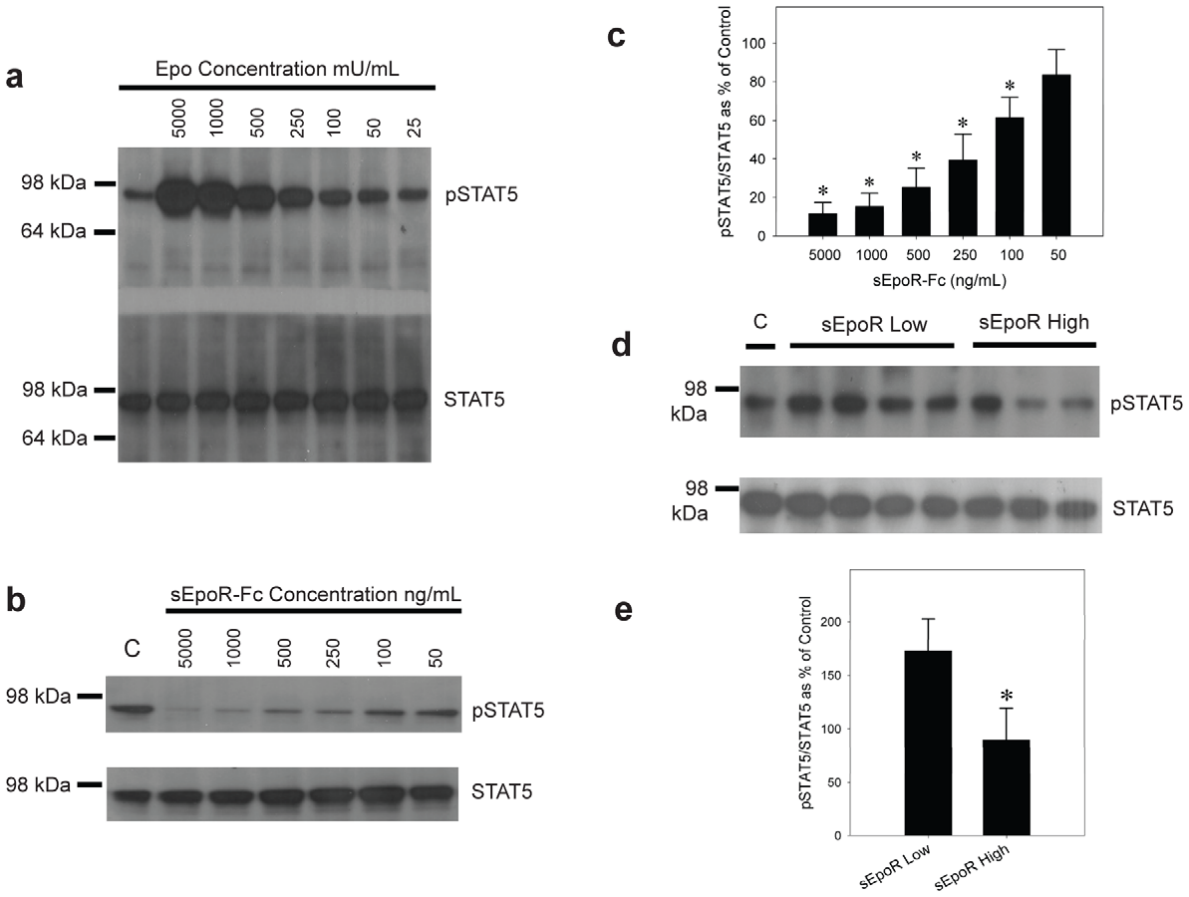
Functional characterization of sEpoR. **3a.** Western blot showing increasedphospho-Stat-5 in the presence of increasing erythropoietin (25 to 5000 mU/ml) in BaF3/EpoR cell lysates. **3b**. Representative western blot showing total phospho-Stat-5 and Stat-5 in the presence of erythropoietin 5000 mU/ml and varying concentrations of recombinant sEpoR-Fc (50 -5000 ng/ml). Phospho-Stat-5 decreases with increasing recombinant sEpoR. **3c**. Quantification of sEpoR-Fc inhibition of phospho-Stat 5 (Figure 3b). Ratios of phospho/total Stat-5 (mean ± SD, n = 3) represented as a percentage of control (Epo alone). *represents p<0.05. **3d**. Serum from patients with high sEpoR blocks erythropoietin mediated Stat-5 phosphorylation. Shown is the ratio of phospho-Stat-5 to Stat-5 as measured by densitometry. Serum starved BaF3/EpoR cells were exposed to vehicle (negative control), erythropoietin at 50 mU/ml (positive control) and erythropoietin plus 10% serum with Low sEpoR (≤62.5 pg/ml) or serum with high sEpoR (≥4000 pg/ml) for 10 minutes. Cells were lysed in RIPA buffer and 10 ug protein/lane was run on a 4-12% denaturing gel. Gels were transferred and blotted with anti-Stat-5 and anti-phospho-Stat 5. **3e**. Quantification of western data (Figure 3d). Ratios of phospho/total Stat-5 (mean ± SD, n = 5 individual patient samples each for low sEpoR and high sEpoR) represented as a percentage of control (Epo alone). *represents p<0.05.

We next sought to test the hypothesis that circulating sEpoR may block erythropoietin mediated signaling via the membrane bound receptor *in vitro*. We selected the BaF3/EPOR cell line which is a murine pro B cell line stably transfected with EPOR. This cell line is well characterized and responds to erythropoietin by increasing intracellular signaling via the Stat-5 and Janus kinase 2 (Jak2) pathways resulting in cell proliferation [35], [36], [45], [46]. Using the BaF3/EpoR cells we established an *in vitro* system in which to test Stat-5 phosphorylation in response to erythropoietin. **Figure 3a** is a dose-response curve demonstrating increased Stat-5 phosphorylation in response to increasing erythropoietin. **Figure 3b** shows that addition of recombinant sEpoR blocks erythropoietin mediated Stat-5 phosphorylation in this system at concentrations ranging from 50 ng/mL to 5,000 ng/mL. Quantification of this inhibition is shown in **Figure 3c** as the ratio of phospho-Stat-5 to total Stat-5.

We next tested whether serum from patients with high levels of sEpoR blocks erythropoietin mediated signaling when compared to serum from patients with low sEpoR levels. For this assay BaF3/EpoR were stimulated with 50 mU/ml erythropoietin in the presence and absence of 10% human serum from patients with high and low levels of sEpoR. **Figure 3d** shows a representative western blot from this experiment. Quantification by densitometry (**Figure 3e**) shows a significant (p<0.05) decrease in Stat-5 phosphorylation when BaF3/EpoR cells were exposed to serum from patients with high (>4000 pg/ml) sEpoR as compared to serum from patients with sEpoR levels ≤62.5 pg/ml).

### sEpoR Is Increased by Pro-Inflammatory Cytokines IL-6 and TNF-α

**Figure 4:**
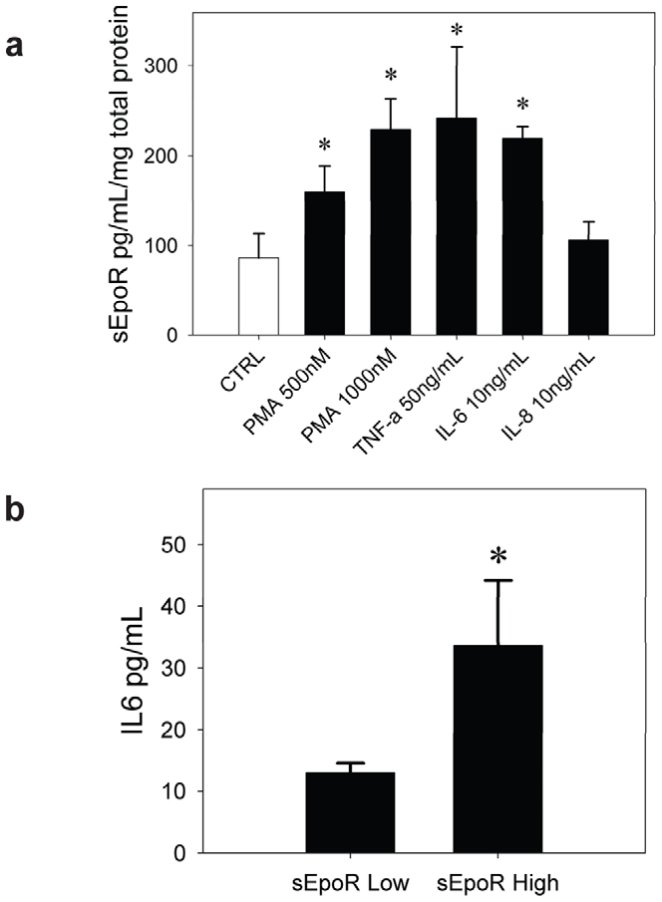
sEpoR is regulated by proinflammatory cytokines. **4a.** IL-6, TNF-α and PMA increase sEpoR in the supernatant of K562 cells. K562 were plated in serum free media and exposed for 48 h to vehicle, PMA, IL-6 and TNF-α. At the end of the incubation cells were pelleted and the supernatant subjected to ELISA for sEpoR. sEpoR measurements were corrected for total protein concentration. * represents p value of <0.05 when compared to the control group. **4b.** Mean IL-6 levels in subjects with low (n = 32) and high sEpoR (n = 32) are shown. * represents p value of <0.05 when compared to low sEpoR group.

The source of circulating sEpoR is also unknown. It is plausible that sEpoR levels are regulated, and that sEpoR has physiologic function in some tissues. Some have noted a positive correlation between sEpoR levels and the degree of erythropoiesis [25] while others have not observed an association [28]. In addition, recent data shows a correlation between elevated TNF-α, IL-6 and IL-8 levels and anemia in patients with chronic kidney disease [47]. We hypothesized that sEpoR may be stimulated by inflammatory cytokines present in the uremic patients. In order to test this hypothesis we selected K562 cells a cell line known to express EpoR [48], [49]. We used phorbol ester (PMA) as a positive control as it has been shown to induce secretion of soluble growth factor receptors in other cell lines [50]. We found that sEpoR in the cell supernatant was increased significantly over baseline by exposure to PMA, IL-6 and TNF-α but not IL-8 (**Figure 4a**). We then measured circulating levels of IL-6 in a subset of patients with high sEpoR and low sEpoR and found that IL-6 levels were on average 2.5 times higher in subjects with high circulating levels of sEpoR than in subjects with low sEpoR levels (**Figure 4b**).

## Discussion

In this study we demonstrate that sEpoR is present in the serum of patients at the initiation of CHD, and that higher levels at the start of dialysis are independently associated with subsequent erythropoietin dose administered in the ensuing 6 months from start of treatment. We also demonstrate that sEpoR is a functional protein and inhibits erythropoietin mediated Stat-5 phosphorylation. We further show that serum with high levels of sEpoR decreases erythropoietin mediated Stat-5 phosphorylation compared with serum from patients with low sEpoR levels suggesting that sEpoR may compete with membrane bound EPOR and inhibit erythropoietin stimulated erythropoiesis. Additionally, we suggest a mechanism for elevated sEpoR by showing that IL-6 and TNF-α can increase sEpoR production in the supernatant of cell lines expressing endogenous EpoR. Importantly, we also demonstrate that subjects with high sEpoR on average had higher circulating levels of IL-6 than subjects with low sEpoR.

The hypothesis that sEpoR may modulate erythropoietin signaling has biologic plausibility for several reasons. First, other investigators have shown that sEpoR may block EpoR signaling in vitro [25], [51]. Second there is recent data to suggest that sEpoR may inhibit erythropoietin signaling *in vivo*. Soluble EpoR is expressed in the brain and is down-regulated in response to chronic hypoxia in a recently described murine model [30]. This down-regulation contributes to increased minute ventilation. Infusion of exogenous sEpoR decreases endogenous erythropoietin signaling and blocks the increase in minute ventilation [30]. Also, transgenic expression of sEpoR can block erythropoietin in a rat model [52]. Furthermore, many cytokines of the superfamily type 1 transmembrane proteins (of which EpoR is a member) are synthesized in soluble forms, and alter native ligand receptor binding [26], [27], [53]. Two other groups have reported sEpoR levels in patients with chronic kidney disease or on dialysis but they were not able to show a physiologic role for this protein [25], [26]. Here we confirm the presence of sEpoR by immunoprecipitation and ELISA of serum and show that serum enriched in sEpoR inhibits erythropoietin mediated Stat-5 phosphorylation suggesting that sEpoR may have a physiologic role rather than being an epiphenomenon related to the degree of erythropoiesis.

We speculated that sEpoR levels may be modulated by cytokines present in the chronic inflammatory state present in many dialysis patients. Data regarding the regulation of sEpoR is limited, although membrane bound EpoR is known to be regulated by several cytokines such as TNF-α, IL-1β, and IL-6 [54], [55]. Hypoxia and erythropoietin may also stimulate EpoR expression. EpoR is regulated by hypoxia-inducible factor 1 [56] and recent data shows increased EpoR expression in primary human venous endothelial cells and bone marrow vascular endothelial cells under hypoxic conditions and in response to erythropoietin [57]. It is unknown if sEpoR is produced in proportion to EpoR of if it is independently regulated. Lysosomal degradation plays an important role in altering the cell surface expression of many cytokine receptors [58]. Interestingly, blocking lysosomal degradation of EpoR with calpain inhibitors increased sEpoR by 2 to 5 fold but only increased membrane bound EpoR 1.5 fold arguing for differential regulation of the two proteins [59]. Here we provide evidence that IL-6 and TNF-α can increase sEpoR in the supernatant of cells known to express the native receptor and that subjects with high sEpoR on average had higher circulating levels of IL-6. This suggests one possible mechanistic link between inflammation and erythropoietin resistance.

Our preliminary data suggest that elevated sEpoR levels at initiation of CHD identify those that require higher erythropoietin doses over the ensuing 6 months. Importantly, sEpoR levels were not correlated with other parameters considered linked to erythropoietin resistance such as ferritin, iron saturation, and parathyroid hormone levels. Higher erythropoietin doses were received in the context of achieving lower hemoglobin values compared to those with lower sEpoR levels. It is interesting to find that mean weekly erythropoietin doses differed most markedly between days 90 and 180. This may be because starting doses of erythropoietin are usually similar when patients initiate CHD (as seen in **Figure 2b**) given the low hemoglobin levels at the initiation of hemodialysis and the ease of administration of standard doses in busy dialysis units. In this context, it is likely that only later is erythropoietin resistance (even in a subtle form) uncovered. Furthermore, since the half-life of red blood cells is 120 days, it is likely that the effects of sEpoR are more prominent at 180 days rather than at 90 days. There are recent data showing that higher erythropoietin dose is associated with increased mortality, and a subset of patients require significantly higher amounts of erythropoietin to sustain adequate hemoglobin levels [60]. It is not clear if this is related to erythropoietin itself, or is associated with risk factors such as chronic inflammation. For this study we chose to closely examine the link between sEpoR levels and subsequent erythropoietin dose, hence we required all subjects to remain alive during the study period. Future studies should examine whether there may be a link between sEpoR levels and outcomes including death.

In this initial description sEpoR levels independently identified those that required higher doses of erythropoietin in the ensuing 6 months even after accounting for standard baseline clinical characteristics and laboratory measures. In these analyses we controlled for routinely measured factors previously linked with erythropoietin resistance, including PTH, iron status, and measures of inflammation such as ferritin. However, we did not measure novel circulating markers associated with erythropoietin resistance such as pro-hepcidin which may be a marker for inflammation and alter iron availability [13]–[15], soluble transferrin receptor which has been shown to be decreased in chronic kidney disease [16]–[19], and C-reactive protein (CRP). Patients with elevated CRP require higher erythropoietin dose and there may be decreased clearance of inflammatory cytokines such as TNF-α, IL-1 and IL-6 [9], [61]. At high levels these cytokines can blunt erythropoietin action on bone marrow precursor cells [10]. It is also possible that some inflammatory cytokines may regulate sEpoR production themselves or they may both be associated with a common factor. Our *in vitro* data suggests that IL-6 and TNF-α may directly stimulate sEpoR production. In future work it would be reasonable to measure pro-hepcidin, soluble transferrin receptor, CRP, IL-6 and TNF-α to see if any of these factors are associated with sEpoR levels.

We studied subjects representative of incident US dialysis patients. However, erythropoietin dosing varies significantly by country with most studies showing the highest doses in the United States [62]. A recent review of erythropoietin dosing patterns in 12 countries showed that average weekly erythropoietin dose among chronic stable dialysis patients in the US was highest at 17,360 IU per week compared with a low of 5,297 per week in Japan [63]. In this study greater erythropoietin dose was associated with greater mean hemoglobin concentration. Our findings would need to be validated in other patient populations, including European and Asian groups to assess generalizability. Our data may have particular relevance to cancer subjects as several tumor cell lines have been shown to secret sEpoR [26] and in whom higher doses of erythropoietin have been associated with mortality [64].

The data presented showing increased sEpoR in response to IL-6 and TNF-α is preliminary. Future work includes a full analysis of sEpoR in response to these and other potential inflammatory cytokines. Questions to be answered include what is the effect on membrane bound EpoR, what is the time course for sEpoR release, and is sEpoR regulated at a translational, transcriptional, or post-transcriptional level? Importantly, we also do not know whether sEpoR may also be involved in the pathogenesis of anemia of chronic disease, which is also thought to be secondary to chronic inflammation [65].

In summary, our findings suggest that sEpoR may play a role in the response of dialysis patients to exogenous erythropoietin and sEpoR may be produced in response to inflammatory cytokines. A better understanding of sEpoR regulation and it association with other factors known to contribute to erythropoietin resistance may lead to more strategic and effective erythropoietin dosing protocols. If sEpoR can be shown in further studies to inhibit erythropoietin effectiveness in dialysis patients then therapeutic strategies aimed at decreasing sEpoR may be useful.
